# Overlapping genes connect rheumatoid arthritis and head and neck cancer: coincidence or shared immune pathophysiology?

**DOI:** 10.3389/fmed.2025.1578016

**Published:** 2025-06-18

**Authors:** Ran Wang, Haiyang Li, Yifan Yang, Meng Lian

**Affiliations:** ^1^School of Pharmacy, Key Laboratory of Ethnomedicine of Ministry of Education, Minzu University of China, Beijing, China; ^2^Department of Otorhinolaryngology Head and Neck Surgery, Beijing Tongren Hospital, Capital Medical University, Beijing, China; ^3^Key Laboratory of Otorhinolaryngology Head and Neck Surgery (Capital Medical University), Ministry of Education, Beijing, China

**Keywords:** rheumatoid arthritis, head and neck cancer, AI-driven computational process, protein–protein interaction, pathways, genetic mechanisms

## Abstract

**Introduction:**

Despite advances in understanding the pathophysiology of rheumatoid arthritis (RA) and head and neck cancer (HNC) individually, their shared genetic and molecular mechanisms remain poorly defined.

**Methods:**

This study aimed to explore gene-level connections between RA and HNC. A comprehensive literature mining approach identified gene–disease associations from PubMed and bioinformatics databases, covering 19,924 genes. An AI-driven computational pipeline applied the Adjusted Binomial Method Algorithm (ABMA) to assess association reliability. Overlapping genes were analyzed through protein–protein interaction (PPI) networks, functional annotation, and literature-based pathway analyses to elucidate common and distinct mechanisms.

**Results:**

The analysis identified 3,697 RA-related and 6,249 HNC-related genes, supported by 13,555 and 16,096 references, respectively, with a significant overlap of 2,549 genes (OR = 7.52; *p* < 1 × 10^−16^). Statistical refinement yielded 224 significant RA genes and 421 significant HNC genes, including 35 overlapping genes (OR = 9.27; *p* = 1.63 × 10^−20^), which formed a dense PPI network (206 edges; density = 0.17; clustering coefficient = 0.67). Seven key hub genes— TLR2, RAC1, RELA, CTSK, CDC42, CXCL11, and CYP2C19—emerged as central nodes in immune and inflammatory regulation. Functional enrichment analysis identified nine significantly enriched pathways or categories, including inflammatory response, chemotaxis, and the chemokine signaling pathway. Pathway analysis further revealed a bidirectional regulatory loop linking RA and HNC via five of these hub genes (RELA, CDC42, CTSK, CXCL11, and CYP2C19), which mediate feedback mechanisms in immune–inflammatory signaling.

**Conclusion:**

These findings highlight robust immuno-inflammatory mechanisms that may serve as shared therapeutic targets for both conditions.

## Introduction

Rheumatoid arthritis (RA) is a chronic autoimmune disorder characterized by joint inflammation, pain, swelling, and potential joint damage. It affects 0.5–1% of the global population, with a higher prevalence in women and typically presenting between ages 40 and 60 (1). RA has a complex etiology involving non-modifiable risk factors—such as female sex, family history, and the HLA-DRB1 “shared epitope” genotype—and modifiable factors including cigarette smoking, which can increase RA risk two-to threefold, as well as obesity and silica exposure (1, 2).

Head and neck cancer (HNC) encompasses malignancies arising in the oral cavity, pharynx, larynx, and related structures. Head and neck squamous cell carcinoma (HNSCC), which accounts for approximately 90% of all HNCs, is the seventh most common cancer worldwide, with an estimated 890,000 new cases and 450,000 deaths each year—representing about 4.5% of all global cancer diagnoses and deaths (3). In the United States, HNSCC constitutes around 3% of all cancers. Major risk factors include tobacco smoking and alcohol consumption, which together account for up to 90% of cases (4). Additionally, high-risk human papillomavirus (HPV) infection is a key driver of the increasing incidence of oropharyngeal cancers (5). Other contributing factors in certain populations include poor oral hygiene, betel quid chewing, and occupational exposures such as wood dust and formaldehyde (6). Emerging clinical and epidemiological data reveal a surprising comorbidity between RA and HNC, suggesting they share core molecular drivers of immune dysregulation and chronic inflammation (7, 8). Population studies report an elevated incidence of RA among HNC survivors—particularly middle-aged men with oral cancer—underscoring a bidirectional risk that clinical factors alone cannot explain (8).

At the cellular level, both RA synoviocytes and cancer-associated fibroblasts undergo metabolic reprogramming, shifting toward glycolytic and lipid-utilization phenotypes that sustain inflammation and tissue remodeling (9, 10). These convergent pathways point to shared genetic susceptibilities—such as polymorphisms in cytokine signaling or antigen-presentation genes—that may predispose individuals to both autoimmunity and oncogenesis. Mapping these gene-level connections could therefore illuminate molecular crosstalk, identify cross-disease biomarkers, and guide the development of therapies targeting common inflammatory and cancer pathways.

In both RA and HNC, inflammation acts as a double-edged sword, driving autoimmune responses in the former and promoting tumor growth and metastasis in the latter. Mechanistic studies have highlighted shared processes such as immune checkpoint dysregulation, oxidative stress, and metabolic reprogramming. For instance, somatic mutations in STAT3, frequently associated with large granular lymphocyte leukemia in RA patients, have been implicated in cancer pathways (11). Moreover, the PD-1/PD-L1 immune checkpoint axis, a therapeutic target in cancer immunotherapy, also plays a role in modulating T-cell responses in RA (12). Genetic predispositions, such as those influencing alcohol metabolism, further illustrate the complex relationship between these conditions, as alcohol consumption is linked to a reduced risk of RA but an increased risk of HNC (13).

Despite advances in understanding the pathophysiology of RA and HNC individually, the shared genetic and molecular mechanisms linking these diseases remain poorly understood. This study aims to explore the genetic overlap between RA and HNC, including its major subtype oral squamous cell carcinoma (OSCC), by identifying significant shared genes, pathways, and hub genes using AI-driven literature mining, the Adjusted Binomial Method (ABM), and gene expression validation. We hypothesize that these overlapping genetic mechanisms contribute to the interconnected pathophysiology of RA and HNC/OSCC, potentially revealing novel therapeutic targets. By investigating these shared pathways and gene networks through bioinformatics and network-based analyses, we aim to provide new insights into the interplay between autoimmune inflammation and cancer, ultimately paving the way for innovative strategies to address both conditions.

## Materials and methods

### Study workflow

The workflow involved four main steps: (1) A comprehensive literature mining effort was undertaken to gather scientific reports identifying potential relationships between 19,924 genes and two diseases—Rheumatoid Arthritis (RA) and head and neck cancer (HNC). (2) An AI-driven computational approach was then applied to construct a relationship table, using the Adjusted Binomial Method Algorithm (ABMA) to evaluate the reliability of each gene-disease relationship; to control for false discoveries, a False Discovery Rate (FDR) correction was applied. (3) Gene lists associated with each of the two diseases were compared to identify unique and overlapping genes across RA and HNC. (4) For overlapping genes, functional pathway analysis, protein–protein interaction (PPI) analysis, and literature-based pathway analysis were performed to explore gene-level connections among the three diseases.

### Disease gene identification using literature-based mining

A comprehensive literature mining effort was conducted to identify potential relationships between whole-genome genes (19,924 genes) and two diseases: RA, and HNC. This process involved using the Entrez API^1^ to retrieve disease-gene references from PubMed^2^ and the AIC Bioinformatics Toolbox (ABT) to gather data from the AIC Bioinformatics Database (ABD).^3^ Relevant reference information, including the title, publication date, PMID, DOI, abstract, and other details, was organized into an Excel worksheet for post-processing and further analysis. The Entrez Programming Utilities (E-utilities) are a set of server-side programs provided by the National Center for Biotechnology Information (NCBI). They offer a stable interface to the Entrez query and database system, allowing for programmatic access to various NCBI databases, including PubMed, Gene, and Protein. This facilitates automated retrieval of biomedical literature and data for large-scale analyses.^4^ The ABT is an AI-driven platform that leverages the AIC Bioinformatics Database (ABD) to extract and analyze data across various domains within biology and bioinformatics. It employs natural language processing methods to process literature from sources like PubMed, arXiv, and bioRxiv, enabling comprehensive literature mining and data integration (see text footnote 3).

### AI-based relationship table construction

To construct a high-confidence table of gene–disease relationships, we implemented a multi-stage, AI-driven extraction and quality control pipeline, developed in-house and based on the ChatGPT API (model GPT-4o). This system was designed to extract relevant biomedical relationships and assign confidence scores, directionality, and polarity. The process included the following key components:

1. Information extraction and summarization

From each literature reference, the AI system extracted sentence-level evidence involving co-occurrence of gene and disease terms. We used a trained natural language processing (NLP) module to extract Subject–Predicate–Object triples describing the relationship.

2. Polarity and direction assignment

For each extracted relationship, the predicate (action word or phrase) was compared against a manually curated dictionary of polarity-indicative keywords. For example:

Positive polarity: promotes, activates, induces, upregulates, enhances.

Negative polarity: inhibits, suppresses, downregulates, blocks.

The directionality was defined as Subject → Object, where the subject is the upstream regulator (often the gene) and the object is the downstream entity (e.g., a disease or another gene). For example, in the sentence “Gene A inhibits Disease B,” polarity is negative, and direction is Gene A → Disease B.

3. ChatGPT API-based quality control

To ensure the accuracy of NLP-based polarity and direction assignments, we applied a secondary verification using the ChatGPT API. The model was instructed to evaluate the full abstract or sentence containing the candidate relationship and to confirm whether the extracted polarity and direction were consistent with the context. Discrepancies or ambiguous cases were flagged and either corrected or excluded from further analysis.

### Adjusted binomial method algorithm

Following extraction, we evaluated the reliability of each gene–disease relationship using the Adjusted Binomial Method Algorithm (ABMA). This statistical method considers literature support and polarity agreement across sources to score the confidence of each relationship. To reduce the risk of false discoveries, we applied a False Discovery Rate (FDR) correction, retaining only relationships with a q-value ≤ 0.01. This integrated approach enabled us to generate a refined set of gene–disease relationships that are statistically robust, biologically relevant, and suitable for downstream pathway and network analyses.

The Adjusted Binomial Method algorithm (ABMA) assesses the association between two entities, such as a gene and a disease, using an adjusted binomial test (scipy.stats).^5^ SciPy is an open-source Python library used for scientific and technical computing. The scipy.stats module within SciPy offers a wide range of statistical functions, including the binomial test, which is used to assess the significance of observed frequencies against expected probabilities. This is particularly useful in evaluating the reliability of gene-disease associations identified through literature mining.^6^

This ABMA method considers the results of multiple different observations, including positive, negative, and inconclusive findings, and assesses the association between two entities by determining if the observed proportion of a dominant results (e.g., positive association) significantly differs from a hypothesized probability (p0). Here’s a breakdown of the statistical steps and equations involved:

### Total observations calculation

To ensure a statistically sound evaluation of gene–disease associations derived from heterogeneous literature sources, we calculated the total number of effective observations (N) by accounting for both reported and potentially missing studies. This step is essential because literature mining may yield varying levels of evidence (positive, negative, or unknown), and overlooking the possibility of publication bias or unreported findings could compromise the statistical inference. Incorporating an estimate for uncovered studies enhances the robustness of the Adjusted Binomial Method Algorithm (ABMA), especially in large-scale, text-mined datasets where data completeness cannot be guaranteed (14).

The total sample size N is calculated using the following formula:


$$N=n_{p}+n_{n}+n_{0}+n_{x}$$

Where, N represents the total effective sample size, $n_{p}$ , $n_{n}$ , and $n_{0}$ represent the sample sizeof positive, negative, or unknown relationship, and $n_{x}$ represent uncovered samples. To account for potential publications not identified through the initial search, we use an uncovered sample fraction factor α , which represents the ratio of uncovered to covered samples:


$$n_{x}=\alpha *(n_{p}+n_{n}+n_{0})$$

For this study, the fraction factor α is set to 1. This choice is based on the assumption that PubMed and the ABD database together provide comprehensive coverage of bioinformatics and biology studies, capturing around 50% of publications in the field. Therefore, assuming uncovered samples to be at most equal to identified samples is a reasonable estimate.

### H0 testing using adjusted binomial test

*Null hypothesis (H0)*: The true proportion of dominant results is equal to p0.

*Alternative hypothesis (H1)*: the true proportion of dominant results (e.g., positive associations) is greater than p0.

*Decision rule*: To evaluate the significance of a dominant relationship polarity (e.g., a positive association between a gene and disease), we use a one-tailed binomial test. The decision rule is as follows: if the computed *p*-value is less than or equal to the significance level (*α* = 0.05), we reject the null hypothesis, indicating that the observed polarity is statistically dominant.

The *p*-value was calculated using the function below:


$$\;p-\text{value}=P(X\geq n_{p})=\text{binom}.\mathrm{sf}(n_{p}-1,N,p0)$$

Where binom.sf is the survival function of the binomial distribution, n_p_ is the observed number of dominant polarity findings, N is the total number of adjusted observations, and p₀ is the null proportion.

To account for potential noise and ambiguity in the literature, we adjust the total number of observations using:


$$N=2*(n_{p}+n_{n}+n_{0})$$

With the number of dominant findings is then:


$$n_{p,\mathrm{adj}}=2*n_{p}$$

The observed proportion of dominant findings is given by:


$$\;n_{\text{obser}}=\frac{n_{p,\mathrm{adj}}}{N}\in (0.33,1].$$

This range reflects the condition that the dominant polarity must occur more frequently than either of the other two polarity types. Based on this, we set the threshold p₀ = 0.34 to represent the minimum proportion needed for a polarity to be considered dominant under the alternative hypothesis.

### Gene comparison across diseases

The gene lists associated with each of the two diseases—RA and HNC—were compared to identify unique and overlapping genes. Fisher’s exact test was used to assess the significance of the overlap, and a Venn diagram was employed for visualization. While we compared both all disease-related genes and those that were statistically significant, our subsequent analysis will focus primarily on the genes showing statistical significance.

To further explore disease heterogeneity within head and neck cancer (HNC), we conducted an additional gene-level comparison between rheumatoid arthritis (RA) and oral squamous cell carcinoma (OSCC), a major subtype of HNC. The same literature-mining and statistical filtering pipeline was used. Significance of gene overlap was assessed using Fisher’s exact test (q ≤ 0.01), and the findings are presented in Supplementary Tables 1, 4.

### Functional analysis of overlapping genes

For overlapping genes, functional annotation analysis was conducted to understand the roles of genes shared by the two diseases—RA and HNC. This analysis used the “Functional Annotation Tool” of Database for Annotation, Visualization, and Integrated Discovery (DAVID)^7^ to assess gene function across three gene ontologies (GOTERM_BP_DIRECT, GOTERM_CC_DIRECT, and GOTERM_MF_DIRECT) and three pathways (BBID, BIOCARTA, and KEGG_PATHWAY). DAVID provides a comprehensive set of functional annotation tools for investigators to understand the biological meaning behind large lists of genes. It offers functionalities such as gene ontology enrichment analysis, pathway mapping, and clustering of functionally related genes.^8^

Additionally, a protein–protein interaction (PPI) analysis was performed to explore functional connections between these genes. The PPI network was constructed using experimentally validated or literature-supported interactions, and its topological properties were analyzed to assess network structure and identify key hub genes. The following metrics were evaluated: (1) Network density: the ratio of observed edges to all possible edges, indicating the level of connectivity. (2) Average path length: the average number of steps along the shortest paths between all pairs of genes, reflecting how easily signals or effects may propagate through the network. (3) Clustering coefficient: the extent to which nodes tend to form tightly knit groups, suggesting local modular structure. (4) Diameter: the length of the longest shortest path in the network, indicating overall compactness. (5) Centrality measures: Degree centrality identifies genes with many direct connections. (6) Betweenness centrality captures genes acting as bridges in shortest paths. (7) Eigenvector centrality reflects gene influence based on the importance of its neighbors.

These metrics were used to identify hub genes—genes with high centrality across multiple metrics—which are likely to play central roles in disease mechanisms. The biological significance of these hub genes was further examined via functional enrichment analysis.

### Construction of directed pathway connecting RA and HNC

To investigate potential mechanistic links between rheumatoid arthritis (RA) and head and neck cancer (HNC), we constructed a directed pathway based solely on literature-derived gene–disease associations extracted using our AI-based pipeline. We identified genes with statistically significant (q ≤ 0.01, after FDR correction) directional relationships with both RA and HNC, as determined by the Adjusted Binomial Method Algorithm (ABMA).

For each selected gene, two directional associations were required: one from RA to the gene (RA → gene) and one from the gene to HNC (gene → HNC), or vice versa. These associations were used to construct directed paths such as RA → gene → HNC or HNC → gene → RA. Importantly, non-directional or convergent relationships—such as RA → gene ← HNC—were excluded, as they do not form a valid causal or regulatory path within a directed network.

Each relationship included polarity (positive, negative, or neutral) and directionality, as assigned by our natural language processing system and verified through ChatGPT-based quality control. The resulting RA–gene–HNC triplets formed the basis for constructing a bidirectional regulatory framework. A detailed presentation of these pathways is provided in the “Results” section.

## Results

### AI-based disease-gene identification results

**Figure 1:**
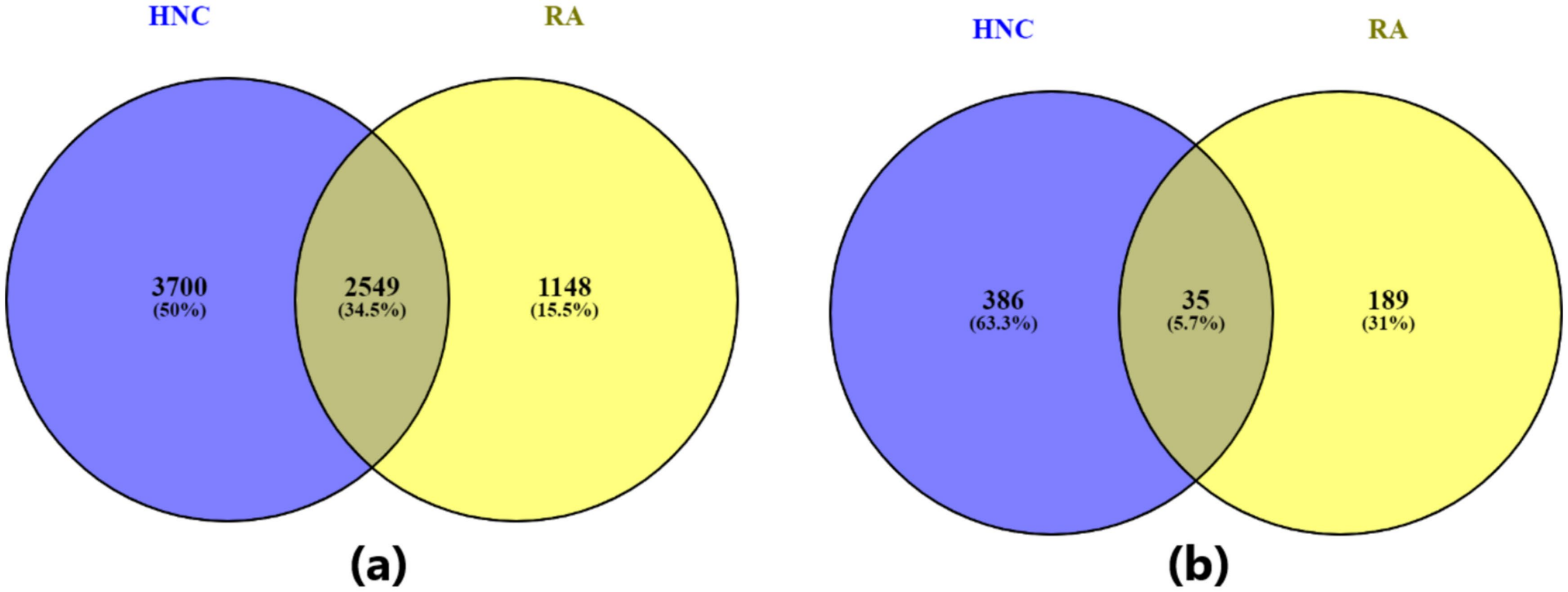
Venn diagram illustrating the overlap between genes associated with the two diseases—rheumatoid arthritis and head and neck cancer. **(A)** Venn diagram based on all identified disease-related genes; **(B)** Venn diagram based on statistically significant disease-related genes (*q*-value ≤ 0.01).

Out of a total of 19,924 genes, the AI-based computational approach identified 3,697 genes associated with rheumatoid arthritis (RA), supported by 13,555 references, and 6,249 genes associated with head and neck cancer (HNC), supported by 16,096 references. Among these, 2,549 genes were found to overlap between RA and HNC. As shown in Figure 1A, this overlap is statistically significant, with a Fisher’s exact test yielding an odds ratio (OR) of 7.52 and a *p*-value <1E-16 (Table 1), indicating a strong enrichment of shared genes between the two diseases.

**Table 1:** Venn diagram statistics for overlapping genes among two diseases.

Gene category	Source disease	Target disease	#Genes source	#Genes target	Overlap	Odds ratio	*p*-value
All genes	HNC	RA	6,249	3,697	2,549	7.52	<1E-16
Significant genes (*q*-value ≤ 0.01)	HNC	RA	421	224	35	9.27	1.63E-20

A *p*-value of <1E-16 indicates an extremely significant overlap, below standard numerical precision thresholds.

As shown in Figure 1B and Table 1, when a stricter significance threshold was applied (*q* < 0.01), 224 RA-associated genes and 421 HNC-associated genes were retained, with 35 overlapping genes. This refined overlap remained statistically significant, with an odds ratio of 9.27 and a p-value of 1.63E-20, further confirming the robustness of the shared genetic association. These findings underscore a potential common molecular basis linking RA and HNC and support the hypothesis of shared genetic pathways underlying these conditions.

To address the heterogeneity within HNCs, we performed an additional analysis specifically evaluating shared genes between RA and oral squamous cell carcinoma (OSCC). Among the 198 RA-associated and 224 OSCC-associated genes (*q* < 0.01), 13 genes overlapped, yielding a statistically significant enrichment (odds ratio = 6.5, *p* = 4e-7). A broader gene-level analysis without q-value filtering revealed 2,499 overlapping genes out of 5,836 (RA) and 3,697 (OSCC), with an even higher odds ratio (8.06) and *p* < 1e-10. Full results are presented in Supplementary Table 1. Additionally, disease–gene relationship data for RA, HNC, and OSCC are provided in Supplementary Tables 2–4.

### PPI analysis

**Figure 2:**
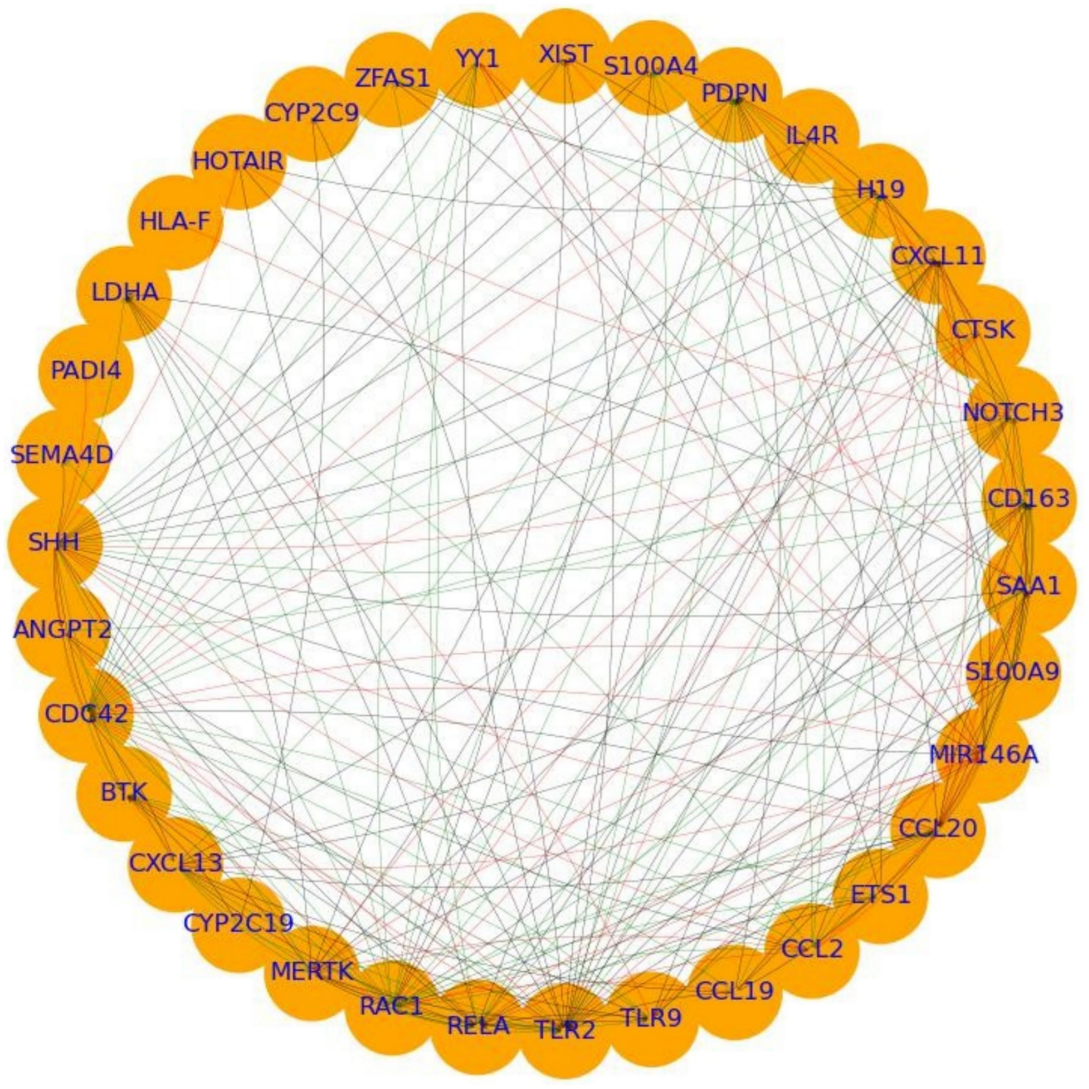
PPI analysis showing the interplay between the overlapping genes between rheumatoid arthritis (RA) and head and neck cancer (HNC).

PPI network analysis of overlapping genes between RA and HNC revealed a moderately dense and cohesive network of 35 genes (ANGPT2, BTK, CCL19, CCL2, CCL20, CD163, CDC42, CTSK, CXCL11, CXCL13, CYP2C19, CYP2C9, ETS1, H19, HLA-F, HOTAIR, IL4R, LDHA, MERTK, MIR146A, NOTCH3, PADI4, PDPN, RAC1, RELA, S100A4, S100A9, SAA1, SEMA4D, SHH, TLR2, TLR9, XIST, YY1, and ZFAS1) connected by 206 edges. The network had a density of 0.17, average path length of 1.73, clustering coefficient of 0.67, and diameter of 3, forming a single connected component. This compact structure indicates tightly interconnected genes potentially involved in shared biological mechanisms (Figure 2).

Centrality analysis revealed RAC1 and TLR2 as central hubs, with high in-degree (0.29, 0.56), out-degree (0.56, 0.26), betweenness centrality (0.06 each), and eigenvector centrality (0.30, 0.29). MIR146A showed moderate in-and out-degree (0.29, 0.32), elevated betweenness (0.04), and high eigenvector centrality (0.23). CXCL13 and SHH displayed complementary connectivity patterns: CXCL13 had high out-degree (0.41) and eigenvector centrality (0.21), while SHH had the highest out-degree (0.74) and notable eigenvector centrality (0.27). These findings support the classification of TLR2, RAC1, MIR146A, SHH, and CXCL13 as hub genes likely mediating molecular crosstalk between RA and HNC. Functional enrichment of these hubs is presented in the following section.

### Functional annotation analysis results

To investigate the biological relevance of genes shared by rheumatoid arthritis (RA) and head and neck cancer (HNC), functional enrichment analysis was conducted using the DAVID Functional Annotation Tool. This analysis focused on three Gene Ontology categories (GOTERM_BP_DIRECT, GOTERM_CC_DIRECT, and GOTERM_MF_DIRECT) and three pathway databases (BBID, BIOCARTA, and KEGG_PATHWAY).

**Figure 3:**
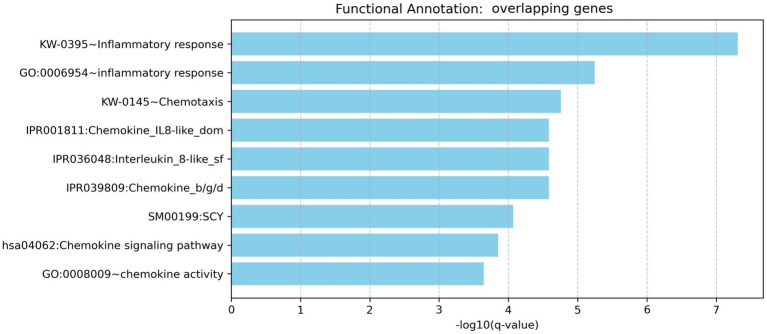
Functional enrichment analysis for overlapping genes associated with both rheumatoid arthritis and head and neck cancer.

The analysis revealed nine significantly enriched pathways or functional categories (Figure 3) by the 35 overlapping genes, with a focus on processes related to inflammatory response, chemotaxis, chemokine activity, and cytokine-related signaling pathways. Key enriched terms included inflammatory response (GO: 0006954, Bonferroni-adjusted *p* = 1.18 × 10^−5^), chemotaxis (KW-0145, Bonferroni-adjusted *p* = 3.50 × 10^−5^), and the chemokine signaling pathway (KEGG hsa04062, Bonferroni-adjusted *p* = 1.76 × 10^−4^). Multiple genes—such as CXCL11, CCL20, CCL19, CCL2, CXCL13, S100A9, TLR2, and RELA—were involved in more than one pathway or GO term, highlighting their pleiotropic roles in immune modulation.

To correct for multiple comparisons, a Bonferroni correction was applied. Given the 35 genes tested, the significance threshold was set at 0.00143 (i.e., 0.05/35). All reported enriched terms met this stringent threshold, reinforcing the statistical robustness of the findings. These results suggest that the overlapping genes are significantly associated with shared immuno-inflammatory mechanisms, potentially linking the pathophysiology of RA and HNC at the molecular level.

### Pathway connecting RA and HNC

**Figure 4:**
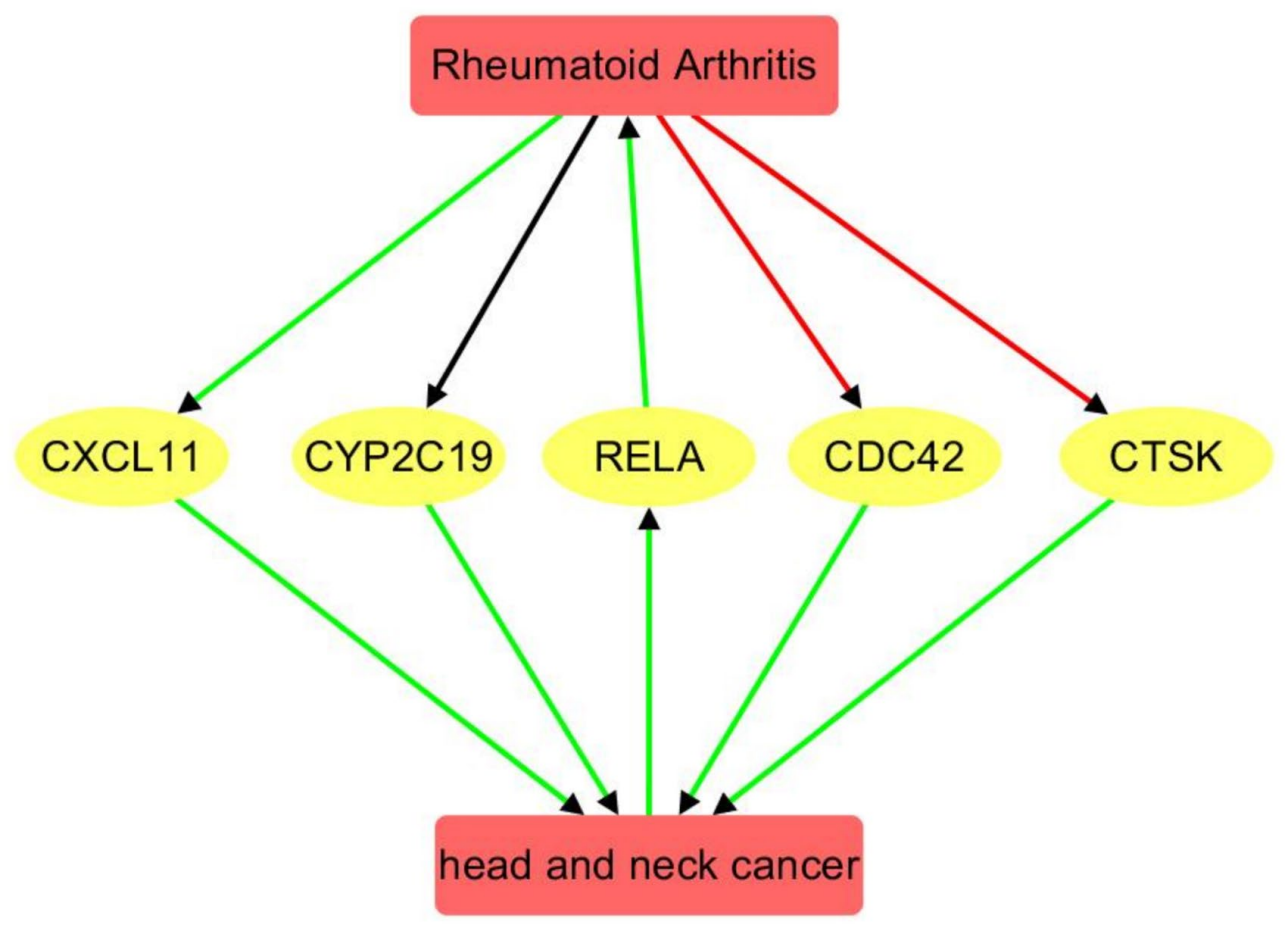
Pathway connecting rheumatoid arthritis and head and neck cancer. Red edge indicates a negative association, green positive association, and black unknown association.

Functional pathway analysis revealed a bidirectional regulatory relationship between rheumatoid arthritis (RA) and head and neck cancer (HNC) mediated through a network of functionally connected genes (Figure 4). HNC positively influences the transcription factor RELA (4 references; *q* = 0.0032), which in turn strongly promotes RA (16 references; *q* = 2.93 × 10^−12^), indicating a highly significant and reinforcing HNC → RELA → RA pathway.

RA regulates several downstream genes with both positive and negative polarity. It negatively regulates CDC42 (8 references; *q* = 6.77 × 10^−4^) and CTSK (15 references; *q* = 5.99 × 10^−4^), while positively influencing CXCL11 (10 references; *q* = 0.0011). The regulation of CYP2C19 by RA appears mixed or neutral (14 references; *q* = 0.0017), suggesting a complex or context-dependent relationship.

These RA-influenced genes, in turn, positively affect HNC: CDC42 (5 references; *q* = 7.74 × 10^−4^), CTSK (4 references; *q* = 0.0032), CXCL11 (4 references; *q* = 0.0032), and CYP2C19 (4 references; *q* = 0.0032). All gene → HNC connections exhibit positive polarity, forming a feedback loop whereby RA modulates genes that subsequently reinforce HNC development. The consistency of positive polarity and significant q-values in the RA → gene → HNC direction underscores a potential molecular link and shared pathological mechanisms between RA and HNC. Supporting references and pathway relationship information corresponding to Figure 4 are provided in Supplementary Table 5.

## Discussion

This study provides a comprehensive systems-level investigation into the molecular interconnection between rheumatoid arthritis (RA) and head and neck cancer (HNC), leveraging an AI-based literature-mining framework integrate with protein–protein interaction (PPI) analysis, functional enrichment, and pathway modeling. Our findings identify and validate a statistically significant genetic overlap between the two diseases, offering novel insights into shared immuno-inflammatory mechanisms and suggesting a bidirectional regulatory relationship between RA and HNC. This study addresses a critical gap by investigating RA and HNC not as isolated diseases, but through their shared genetic and molecular intersections.

Prior epidemiological studies have reported elevated RA risk in patients with HNC—particularly among middle-aged males and oral cancer survivors (8) —yet the molecular mechanisms remained underexplored. Leveraging an AI-driven disease-gene identification approach, our analysis uncovered 2,549 genes shared between RA and HNC out of 3,697 RA-related and 6,249 HNC-related genes. This overlap is highly significant (OR = 7.52, *p* = 0), and remained robust even after applying a stringent significance threshold (q < 0.01), with 35 overlapping genes (OR = 9.27, *p* = 1.63 × 10^−20^). While AI technologies have previously enhanced diagnostics and treatment strategies for both diseases (15, 16), our findings provide a statistically supported perspective on their shared molecular basis. These insights could facilitate the development of integrated diagnostic biomarkers and dual-purpose therapies targeting common pathways in both RA and HNC.

To elucidate the mechanistic basis of the RA–HNC connection, we focused on five key genes—RELA, CTSK, CXCL11, CDC42, and CYP2C19—which form the core of the directed RA–gene–HNC pathway (see “Results”). These genes were selected based on their statistically significant, directionally coherent relationships with both RA and HNC, and their central roles in immune-related regulatory networks.

RELA, a subunit of the NF-κB transcription factor complex, plays a critical role in inflammation, immune activation, and cell survival. In RA, RELA expression is elevated in synovial tissue macrophages, driving persistent NF-κB pathway activation and proinflammatory cytokine release (17, 18). In HNC, overexpression of RELA has been associated with tumor progression, resistance to chemotherapy, and increased invasiveness through pathways such as IKK-NFκB/RELA and USP14-mediated signaling (18).

CTSK (Cathepsin K) is a lysosomal cysteine protease essential for collagen degradation and bone resorption. In RA, CTSK is highly expressed at the pannus–bone interface and contributes to cartilage and bone erosion (19). In HNC, particularly oral squamous cell carcinoma, CTSK is overexpressed in tumor and stromal cells, correlating with lymph node metastasis and poor prognosis (20).

CXCL11 is a chemokine that attracts CXCR3^+^ T cells and is implicated in synovial inflammation in RA. Elevated CXCL11 levels have been detected in RA synovial fluid and are produced by synovial fibroblasts under inflammatory stimulation (21). In HNC, CXCL11 promotes tumor angiogenesis and epithelial–mesenchymal transition (EMT), aiding in metastasis and immune evasion through JAK/AKT and MMP7 activation (22).

CDC42, a member of the Rho family of small GTPases, regulates actin dynamics and cell polarity. In RA, CDC42 levels are inversely correlated with disease activity and inflammation markers such as CRP, ESR, and DAS28 scores, indicating a potential immune-modulatory role (23). In HNC, CDC42 enhances tumor cell migration and invasion by promoting cytoskeletal remodeling and EMT, and elevated levels are associated with tumor aggressiveness (24).

CYP2C19, a cytochrome P450 enzyme involved in drug metabolism, plays a role in the bioactivation of disease-modifying antirheumatic drugs (DMARDs) in RA. Poor-metabolizer genotypes (e.g., CYP2C19*2/3) are associated with altered leflunomide metabolism and increased risk of treatment-related toxicity or therapeutic failure (25). In HNC, CYP2C19 polymorphisms have also been linked to increased cancer susceptibility, with poor-metabolizer alleles (e.g., CYP2C192) showing a significant association with higher risk of developing squamous cell carcinoma (26).

While these five genes were prioritized for detailed discussion due to their clear directional involvement in RA–HNC regulatory pathways, our analysis also identified a broader set of 35 overlapping genes. These include additional hub genes identified through PPI centrality analysis (e.g., TLR2, RAC1, MIR146A, SHH, CXCL13), as well as immune modulators (CCL19, IL4R), noncoding RNAs (HOTAIR, XIST, ZFAS1), and signaling mediators (NOTCH3, ETS1, PDPN). Although not all could be discussed in depth here, they represent functionally diverse components of shared immuno-inflammatory mechanisms and warrant further investigation in future studies.

Systemic inflammation and immune dysregulation characteristic of RA may contribute to HNC development through several cellular mechanisms. Chronic inflammation marked by elevated CRP and ESR fosters a tumor-supportive microenvironment (27). Angiogenesis links RA and HNC by supporting pannus formation and tumor growth, respectively (28, 29). The Jak/STAT pathway, crucial in RA inflammation, may further promote a tumor-friendly environment, increasing HNC risk (30).

At the tissue and organ levels, RA and HNC intersect notably in the oral mucosa, where RA-associated periodontitis may elevate oral cancer risk (31). Salivary autoantibodies indicate mucosal immune responses potentially influencing cancer progression (32). Autoimmune thyroid disease often co-occurring with RA may exacerbate symptoms and contribute to thyroid malignancies, a subset of HNC (33). Additionally, lymphatic system alterations in RA may facilitate HNC metastasis (34, 35). These findings underscore the interconnected pathophysiology of RA and HNC and highlight the importance of integrated clinical screening and therapeutic strategies.

Our findings also extend to the subtype level, with a focused analysis revealing a significant gene-level overlap between RA and oral squamous cell carcinoma (OSCC), a prominent subtype of HNC. This result aligns with emerging clinical and epidemiological evidence linking chronic inflammation in RA to increased OSCC risk (36). Mechanistically, both diseases share dysregulated pathways such as matrix remodeling and chemokine signaling: RA synovial fibroblasts and OSCC tumor cells overexpress matrix metalloproteinases that degrade extracellular matrices and promote invasion (37, 38). Similarly, both RA and OSCC involve chemokine-driven immune cell infiltration, fueling inflammation or tumor immune evasion (39, 40). Notably, CTSK, a lysosomal protease involved in bone erosion in RA, is also overexpressed in OSCC and correlates with lymph node metastasis and poor prognosis (20, 41). These shared mechanisms underscore the importance of OSCC as a clinical focus for investigating cross-disease inflammatory pathways and targeted interventions.

Our study effectively utilizes an AI-based computational approach to identify a significant number of genes associated with RA and HNC, supported by extensive literature evidence. The statistically significant gene overlap, together with PPI network analyses revealing key hub genes, highlights potential therapeutic targets and shared pathways—particularly those related to immune response and inflammation—pivotal for understanding and treating diseases characterized by chronic inflammation and cancer progression. The AI-driven framework offers a scalable and systematic method for uncovering complex disease relationships.

However, the study’s reliance on existing literature and computational predictions may limit discovery of novel genes or pathways not yet documented. The AI-based approach may also be influenced by biases inherent in training data, potentially overlooking less-studied genes. Consequently, experimental validation of the identified genes and pathways remains essential for future research.

## Conclusion

This study reveals a significant genetic and molecular overlap between rheumatoid arthritis (RA) and head and neck cancer (HNC), with 35 shared genes forming a tightly connected network enriched in inflammatory and chemokine signaling pathways. Central hub genes—TLR2, RAC1, MIR146A, SHH, and CXCL13—emerge as key regulators. A core regulatory axis involving RELA, CTSK, CXCL11, CDC42, and CYP2C19 highlights bidirectional crosstalk between RA and HNC. These findings suggest common immuno-inflammatory mechanisms and offer potential therapeutic targets, pending further experimental validation.

## Data Availability

The original contributions presented in the study are included in the article/Supplementary material, further inquiries can be directed to the corresponding author.

## Footnotes

^1^ http://www.ncbi.nlm.nih.gov/Entrez/

^2^ https://pubmed.ncbi.nlm.nih.gov

^3^ https://www.gousinfo.com/en/userguide.html

^4^ https://www.ncbi.nlm.nih.gov/books/NBK25501/?utm_source=chatgpt.com

^5^ https://docs.scipy.org/doc/scipy/reference/stats.html

^6^ https://scipy.org/

^7^ https://david.ncifcrf.gov

^8^ https://davidbioinformatics.nih.gov/?utm_source=chatgpt.com

## Supplementary material

Supplementary Table 1 Shared genes between RA and OSCC at different significance thresholds, including overlap statistics (number of overlapping genes, odds ratios, and *p*-values).

Supplementary Table 2 Gene–disease association data for rheumatoid arthritis (RA) used in the analysis, including relationship name, polarity and direction, and supporting reference information.

Supplementary Table 3 Gene–disease association data for head and neck cancer (HNC) used in the analysis, including relationship name, polarity and direction, and supporting reference information.

Supplementary Table 4 Gene–disease association data for squamous cell carcinoma (OSCC) used in the analysis, including relationship name, polarity and direction, and supporting reference information.

Supplementary Table 5 Supporting references and pathway relationship information corresponding to Figure 4.
